# Genome-wide identification and expression analysis of the *GASA* gene family in Chinese cabbage (*Brassica rapa* L. ssp. *pekinensis*)

**DOI:** 10.1186/s12864-023-09773-9

**Published:** 2023-11-06

**Authors:** Bingxin Sun, Xianlei Zhao, Jiahui Gao, Jie Li, Yue Xin, Yonghui Zhao, Zhiyong Liu, Hui Feng, Chong Tan

**Affiliations:** Department of Horticulture, Shenyang Agricultural University, 120 Dongling Road, Shenhe District, Shenyang, 110866 China

**Keywords:** Chinese cabbage, *GASA*, Bioinformatics, Hormone response, Stress response

## Abstract

**Background:**

The Gibberellic Acid-Stimulated *Arabidopsis* (*GASA*) gene family is widely involved in the regulation of plant growth, development, and stress response. However, information on the *GASA* gene family has not been reported in Chinese cabbage (*Brassica rapa* L. ssp. *pekinensis*).

**Results:**

Here, we conducted genome-wide identification and analysis of the *GASA* genes in Chinese cabbage. In total, 15 *GASA* genes were identified in the Chinese cabbage genome, and the physicochemical property, subcellular location, and tertiary structure of the corresponding GASA proteins were elucidated. Phylogenetic analysis, conserved motif, and gene structure showed that the GASA proteins were divided into three well-conserved subfamilies. Synteny analysis proposed that the expansion of the *GASA* genes was influenced mainly by whole-genome duplication (WGD) and transposed duplication (TRD) and that duplication gene pairs were under negative selection. Cis-acting elements of the *GASA* promoters were involved in plant development, hormonal and stress responses. Expression profile analysis showed that the *GASA* genes were widely expressed in different tissues of Chinese cabbage, but their expression patterns appeared to diverse. The qRT-PCR analysis of nine *GASA* genes confirmed that they responded to salt stress, heat stress, and hormonal triggers.

**Conclusions:**

Overall, this study provides a theoretical basis for further exploring the important role of the *GASA* gene family in the functional genome of Chinese cabbage.

**Supplementary Information:**

The online version contains supplementary material available at 10.1186/s12864-023-09773-9.

## Background

Gibberellic acid (GA) is a ubiquitous plant hormone that regulates plant growth and development [1]. In particular, DELLA protein is a key factor in the gibberellin signaling pathway that contributes to the regulation of plant growth and development processes, including epidermal hair differentiation [2], flower development [3], anther development and flowering [4], stress response [5], and root growth [6]. Gibberellic acid-stimulated *Arabidopsis* (GASA), which is also known as Snakin, is downstream of DELLA and is a type of cysteine-rich peptide (CRP) [7]. Notably, most *GASA* genes are regulated by GA [8].

In *Arabidopsis*, GASA proteins typically consist of 80–270 amino acids, except AtGASA14, which has a proline-rich protein (PRP) motif in the N-terminal region [9]. The GASA proteins have three different domains: (1) an N-terminal signal peptide with 18–29 amino acids; (2) a highly variable hydrophilic region with 7–31 polar amino acid residues displaying a difference between family members both in amino acid composition and sequence length; and (3) a C-terminal GASA domain consisting of 60 amino acids, typically including 12 cysteine residues. The C-terminal GASA domain is considered a key region for maintaining the spatial structures and functions of the GASA proteins [10, 11]. The tomato gene, *gibberellin-stimulated transcript 1* (*GAST-1*), is the first member of the *GASA* family to be identified [12], and many other genes have been identified in different species thus far [13–29]. With the identification of members of the *GASA* gene family across different species, the functions of the gene family have also been comprehensively mapped.

The *GASA* gene family is involved in the regulation of plant growth and development. In *Arabidopsis*, *AtGASA4* regulates branching, floral meristems, floral organ identity, and seed growth [30, 31]. *AtGASA5* is a negative regulator of flowering and stem growth [32]. *AtGASA6* can affect flowering, cell elongation and seed germination [33, 34]. *AtGASA10* also affects anther and seeds [35]. *AtGASA14* controls blade expansion [9]. The *GIP* (encoding the *Petunia hybrida* GA-induced protein), a homolog of *GAST-1*, inhibits flowering and stem elongation [36]. The Gerbera *GEG* gene can regulate cell elongation and petal development [37]. The rice gene, *OsGASR*, and the wheat gene, *TaGASA7*, can regulate grain size and length [38, 39]. *OsGASR1* and *OsGASR2* can affect panicle differentiation in rice [18], and *TaGASR34* can affect the dormancy and germination of wheat seeds [40]. In maize, the *GASA* gene family can affect lateral root development [20, 41]. Silencing the potato’s *snakin-1* gene affects cell activity and changes leaf morphology [42]. The *GASA* gene family in apples is involved in flower induction [23]. *VvGASA7* positively regulates seed size and yield [27]. In strawberry, *FaGAST1* and *FaGAST2* synergistically regulate fruit cell development and affect fruit size [19]. In pear, *PpyGAST1* regulates bud dormancy [43]. In the traditional Chinese medicinal plant, *Salvia miltiorrhiza*, *SmGASA4* promotes the development of roots and flowers [25].

The *GASA* gene family also responds to biotic and abiotic stress in plants. The overexpression of *AtGASA5* negatively regulates heat tolerance in *Arabidopsis thaliana* [44], while *AtGASA14* controls plant resistance to abiotic stress [9]. Leaf expression of the *gerbera* gene, *PRGL* (a *GAST1*-like gene), is induced by injury [45]. Rice contains multiple *GASA* genes that respond to abiotic stress [38]. *TaGASR1* improves wheat tolerance to heat and oxidative stress [46]. The overexpression of *snakin-1* increases potato resistance to fungal and bacterial diseases, and these findings have been verified in lettuce, tomato, and *Peltophorum dubium* [47–50]. The *GASA* gene in rubber tree plays a role in fungal pathogen resistance [24].

Although many studies have been conducted on *GASA* genes across various species, such studies have not been conducted in the context of the Chinese cabbage. The Chinese cabbage is a nutrient-rich cruciferous crop that originates from China. Evolutionarily, the Chinese cabbage is closely related to the model plant, *A. thaliana*. Fifteen *GASA* genes have been identified in *A. thaliana*, and their functions have been verified [34]. Thus, this study was based on the homology between Chinese cabbage and *A. thaliana*. Briefly, herein, we identified members of the *GASA* gene family at the level of the whole-genome of Chinese cabbage and conducted detailed bioinformatics analysis, including chromosome location and gene structure, sequence homology, evolutionary history, synchrony analysis, cis-acting element analysis, protein structure analysis, and subcellular localization. In addition, expression differences and stress responses of the members of the *GASA* gene family in different parts of Chinese cabbage were clarified, laying a foundation for further studies of *GASA* family genes in Chinese cabbage.

## Results and discussion

### Genome-Wide identification and protein features of *GASA* genes in Chinese cabbage

**Fig. 1:**
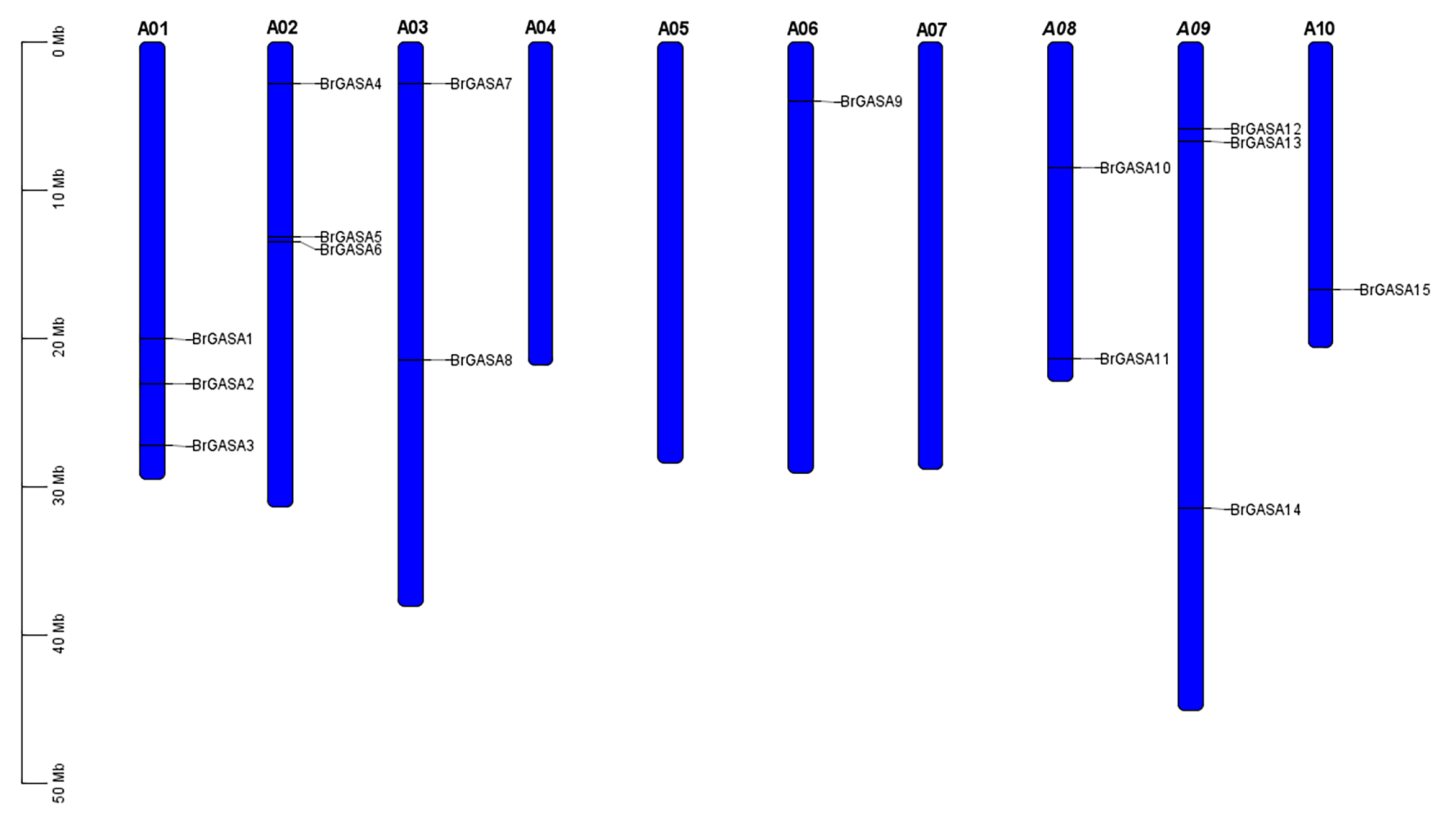
Positions of *GASA* genes on Chinese cabbage chromosomes

**Fig. 2:**
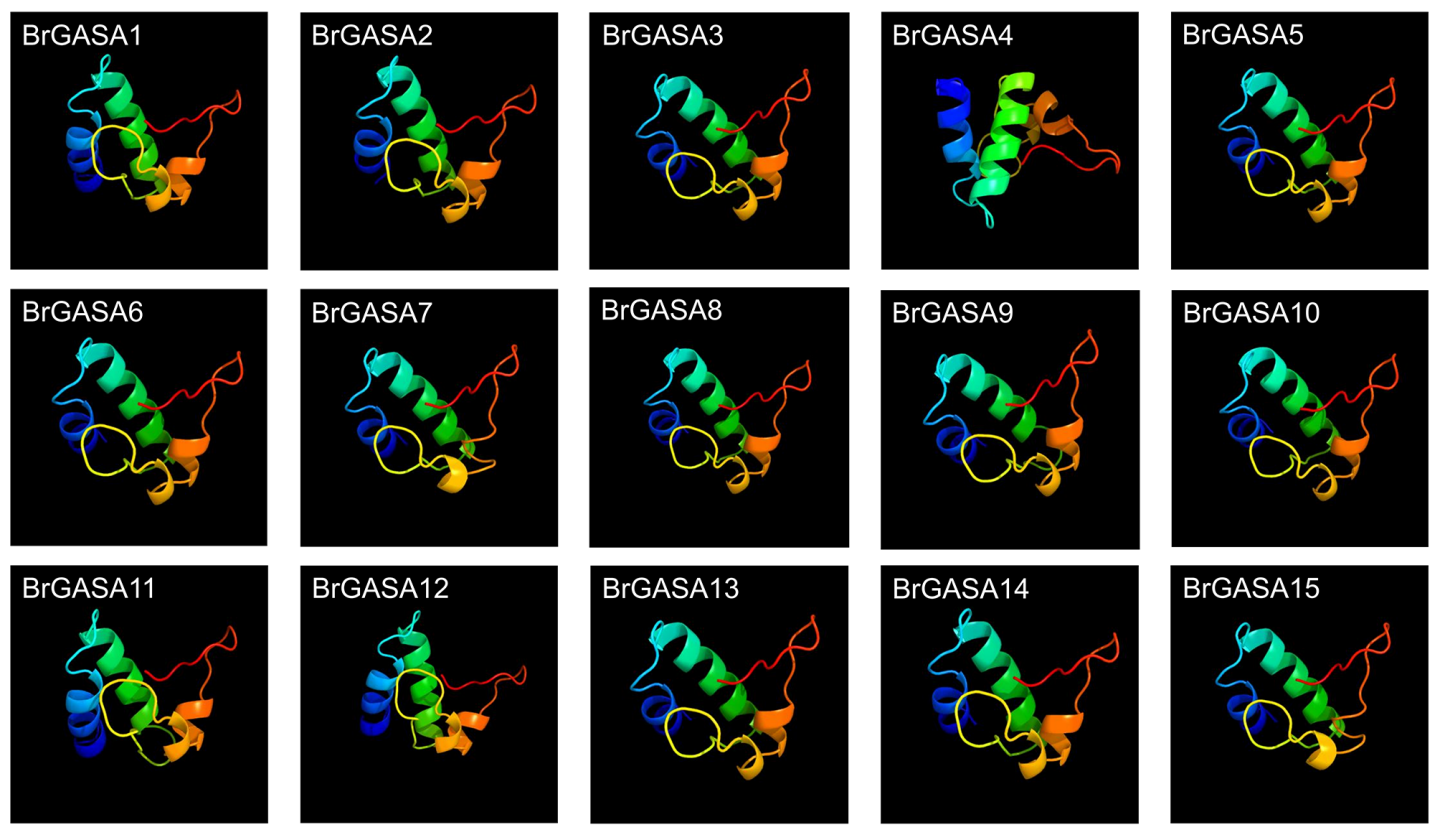
Predicted three-dimensional structures of GASA proteins in Chinese cabbage

In this study, 15 *GASA* genes were identified in the genome network of the Chinese cabbage (Table 1). They were named *BrGASA1* to *BrGASA15*, according to the top-to-bottom position of chromosomes A01-A10. *BrGASA* genes were unevenly distributed across seven chromosomes of the Chinese cabbage genome (Fig. 1). Specifically, three *BrGASA* genes were found on chromosomes A01, A02, and A09; two on chromosomes A03 and A08; and only one *BrGASA* gene was found on chromosomes A06 and A10. In previous reports, the *GASA* genes were found to be randomly distributed in the chromosomes of species such as *Arabidopsis* [23], whereas they were found to be unevenly distributed in those of potato, apple, grapevine, *Zea mays*, *Glycine max*, *Populus* [20, 23, 26–28, 42]. Previous reports have shown that the *Brassica* ancestors experienced extensive gene loss after the Whole-Genome Triplication (WGT) event [51]. Therefore, we believe that the uneven distribution of GASA gene on the chromosomes of the Chinese cabbage genome is closely related to gene loss. Protein characteristics, including molecular weight, isoelectric point, instability index, grand average of hydropathicity (GRAVY), major amino acid content, and aliphatic index, were analyzed using the ExPASy program (Table 2). The number of amino acids in the BrGASA protein was between 64 and 283, with *BrGASA7* encoding the longest protein with highest molecular weight (30.17 kDa), and *BrGASA2* encoding the shortest protein with lowest molecular weight (7.14 kDa). The maximum difference in molecular weight between *BrGASA2* and *BrGASA7* suggests that there could be structural and functional differences between these two genes [27]. The average length of the BrGASA proteins was 124 amino acids, whereas the average molecular weight was 13.35 kDa. Overall, BrGASA was a low-molecular-weight protein, consistent with the results of *Arabidopsis* [52]. Furthermore, the isoelectric point ranged from 6.67 (BrGASA1) to 10.14 (BrGASA7), and the instability index ranged from 26.86 (BrGASA2) to 66.48 (BrGASA4). According to the GRAVY values, Chinese cabbage GASA proteins were hydrophilic. Meanwhile, the aliphatic index values of Chinese cabbage GASA proteins ranged from 27.08 (BrGASA2) to 35.57 (BrGASA1). The main amino acid residues of Chinese cabbage GASA proteins were Cys, Lys, and Gly. In apple, the main amino acid residues of GASA proteins were mainly Cys, Lys and Leu [23]. Predicting the subcellular locations of proteins can provide important clues regarding gene function [29]. In our prediction, the Chinese cabbage GASA proteins were mainly located in the extracellular environment, besides Golgi apparatus, chloroplast, cytoplasm, etc. (Table 2). The localization results of GASA proteins in cotton indicated that they were mostly located extracellular, while a few were in the nucleus and plasma membrane [29]. Not all GASA proteins in potato were located extracellular, and the localization results showed differences. The signal of GASA-GFP fusion protein in rubber tree showed that all proteins could signal in the cytoplasm [24], while GASA protein in *Populus* was found in four positions: Golgi apparatus, cell wall, cell membrane and nucleus [28]. The tertiary structure of proteins facilitates the accurate characterization of protein functions [28]. Based on predictions of the tertiary spatial structures of the Chinese cabbage *GASA* gene family, we found that the protein structures mainly consisted of random coils and α-helix composition, but the β-fold structures were also present (Fig. 2). The similar structural characteristics have also been found in the GASA proteins of apple, grape, poplar, and cotton [23, 27–29].

**Table 1:** Detailed information on *GASA* genes in Chinese cabbage

Gene Locus ID	Gene ID	Chromosome	Start Site	End Site	CDS (bp)	ORF(aa)
*BraA01g030830.3.5 C*	*BrGASA1*	A01	20,052,495	20,053,076	315	104
*BraA01g034960.3.5 C*	*BrGASA2*	A01	23,031,883	23,032,074	192	63
*BraA01g041680.3.5 C*	*BrGASA3*	A01	27,258,165	27,258,826	387	128
*BraA02g005980.3.5 C*	*BrGASA4*	A02	2,820,303	2,821,268	321	106
*BraA02g023240.3.5 C*	*BrGASA5*	A02	13,126,467	13,127,254	372	123
*BraA02g023850.3.5 C*	*BrGASA6*	A02	13,506,970	13,507,434	297	98
*BraA03g006590.3.5 C*	*BrGASA7*	A03	2,834,280	2,835,698	849	282
*BraA03g043210.3.5 C*	*BrGASA8*	A03	21,432,680	21,433,470	321	106
*BraA06g007530.3.5 C*	*BrGASA9*	A06	4,145,012	4,145,212	201	66
*BraA08g009950.3.5 C*	*BrGASA10*	A08	8,486,926	8,487,518	318	105
*BraA08g032610.3.5 C*	*BrGASA11*	A08	21,375,636	21,376,007	258	85
*BraA09g010560.3.5 C*	*BrGASA12*	A09	5,878,441	5,879,203	324	107
*BraA09g012010.3.5 C*	*BrGASA13*	A09	6,753,454	6,753,952	306	101
*BraA09g042130.3.5 C*	*BrGASA14*	A09	31,493,774	31,494,386	363	120
*BraA10g024440.3.5 C*	*BrGASA15*	A10	16,685,009	16,686,428	753	250

CDS: coding sequence; ORF: open reading frame

**Table 2:** Amino acid composition and physiochemical characteristics of GASA proteins in Chinese cabbage

Gene	MW	PI	Major Amino Acid	Instability Index	Aliphatic Index	GRAVY	Localization Predicted
BrGASA1	11.34	6.67	C(11.5), S(9.6), G(7.7)	50.87	35.57	-0.213	extr., vacu., golg.
BrGASA2	7.14	9.42	C(19.0), K(12.7), R(11.1)	26.86	27.08	-0.721	nucl., mito., extr.
BrGASA3	14.18	8.87	P(12.5), C(9.4), L(7.8)	59.55	28.25	-0.53	extr., nucl., chlo.
BrGASA4	11.7	9.36	C(12.3), G(9.4), K(9.4)	66.48	29.92	-0.331	chlo., extr., vacu.
BrGASA5	14.03	9.1	T(11.4), K(10.6), C(9.8)	44.75	33.76	-0.337	chlo., extr., plas.
BrGASA6	10.59	9	C(12.2), A(9.2), L(9.2)	43.23	28.17	-0.028	extr., vacu.
BrGASA7	30.17	10.14	P(30.9), T(10.3), V(9.2)	55.18	30.16	-0.48	extr., chlo., cyto.
BrGASA8	11.23	8.86	K(11.3), C(11.3), G(10.4)	34.52	31.48	-0.028	extr., vacu., chlo.
BrGASA9	7.17	8.48	C(18.2), K(10.6), G(10.6)	45.29	28.86	-0.618	mito., cyto., extr.
BrGASA10	11.45	6.77	C(11.4), K(8.6), L(7.6)	53.2	30.35	-0.199	extr., mito., vacu.
BrGASA11	9.28	8.23	C(15.3), S(10.6), K(9.4)	49.27	27.42	-0.165	chlo., extr., cyto.
BrGASA12	11.39	8.6	K(12.1), C(11.2), G(11.2)	42.17	30.41	-0.035	extr., chlo., mito.
BrGASA13	10.84	8.99	C(11.9), A(8.9), G(8.9)	38.28	28.06	-0.029	extr., vacu.
BrGASA14	13.19	9.1	S(10.8), C(10), A(9.2)	50.13	29.04	-0.324	extr., ER., plas.
BrGASA15	26.53	10.03	P(29.2), T(12.0), V(10.4)	65.14	29.13	-0.471	extr., chlo., cyto.

MW: molecular weight; PI: isoelectric point; GRAVY: grand average of hydropathicity; Extra: extracellular; Golg: Golgi apparatus; Vacu: vacuoles; Chlo: chloroplast; Cyto: cytoplasm; Mito: mitochondria; Nucl: nucleus; Plas: plastids; ER: endoplasmic reticulum

### Analysis of phylogenetic relationship, Gene structure and conserved motifs of GASA Proteins in Chinese Cabbage

**Fig. 3:**
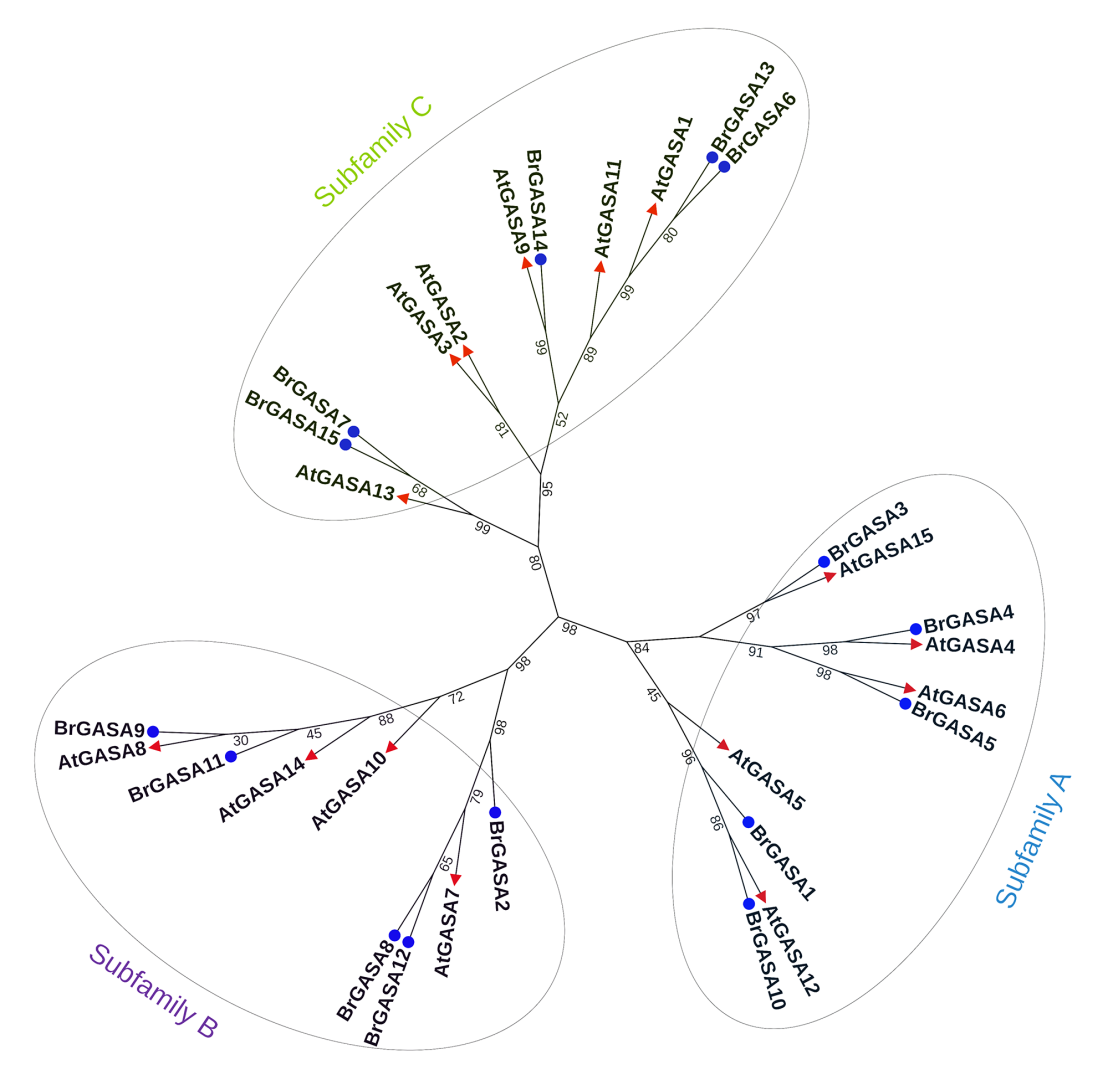
Phylogenetic tree of GASA proteins of Chinese cabbage and *A. thaliana*. Red-colored triangles represent *Arabidopsis* proteins, and blue-colored circles represent Chinese cabbage proteins. Different colored oval shapes indicate different groups

Phylogenetic analysis can help us understand the evolutionary relationships among genes [33]. To classify the *GASA* gene family, we constructed a phylogenetic tree based on the GASA protein sequences of *Arabidopsis* and Chinese cabbage (Fig. 3). The analysis included 30 GASA proteins: 15 from Chinese cabbage and 15 from *Arabidopsis*. There was a similarity between the *GASA* genes of Chinese cabbage and *Arabidopsis*, indicating that these genes might also have functional similarities. As shown in Fig. 3, the proteins were divided into three groups, named subfamily A, B, and C, consisting of 10, 9, and 11 GASA proteins, respectively. Five Chinese cabbage proteins (i.e., BrGASA1, BrGASA3, BrGASA4, BrGASA5, and BrGASA10) and five *Arabidopsis* proteins (i.e., AtGASA4, AtGASA5, AtGASA6, AtGASA12, and AtGASA15) clustered in subfamily A, while five Chinese cabbage proteins (i.e., BrGASA2, BrGASA8, BrGASA9, BrGASA11, and BrGASA12) and four *Arabidopsis* proteins (i.e., AtGASA7, AtGASA8, AtGASA10, and AtGASA14) clustered in subfamily B. Five Chinese cabbage proteins (i.e., BrGASA6, BrGASA7, BrGASA13, BrGASA14, and BrGASA15) and six *Arabidopsis* proteins (i.e., AtGASA1, AtGASA2, AtGASA3, AtGASA9, AtGASA11, and AtGASA13) clustered in subfamily C. In previous studies, *Arabidopsis* GASA proteins were divided into three subfamilies based on its own homology [52]. Subsequently, phylogenetic trees constructed by other species based on the *Arabidopsis* GASA proteins were also divided into three subfamilies, such as maize, rice, apples, wheat, soybeans, grapes, and poplars [20, 23, 26–28, 40].

**Fig. 4:**
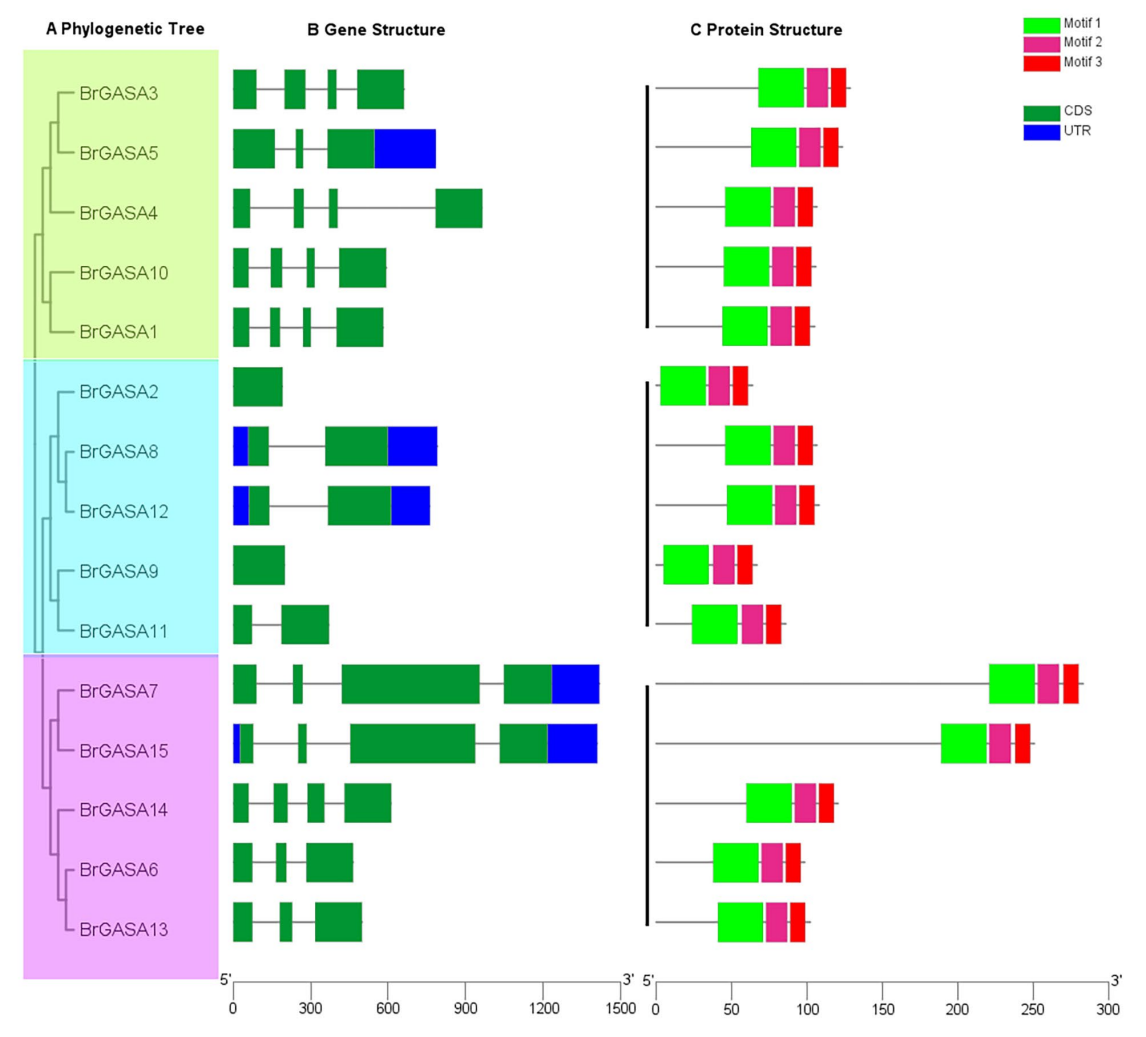
Gene structure and protein motif analysis of the *GASA* gene family in Chinese cabbage. (**A**) Phylogenetic tree of BrGASA proteins. Subfamily A/B/C are represented by green, blue, and pink, respectively. (**B**) Gene structure of *BrGASA* genes. Exons are represented by green boxes, Untranslated regions (UTRs) are indicated by blue boxes and introns by grey lines. (**C**) Conserved motifs of BrGASA proteins. Conserved motifs are represented in different colored boxes. The length of each nucleotide sequence or protein sequence can be estimated using the scale below the picture

An unrooted tree was constructed to explore further the phylogenetic relationships of the GASA proteins in the Chinese cabbage. Similar to the above-described phylogenetic tree data, the Chinese cabbage GASA proteins were divided into three groups in this new analysis (Fig. 4A). The number, location, and length of the exons are closely related to gene homology, and research on exons and introns indicate differences in structure and function between genes [28, 53]. To illustrate the diversity of *GASA* genes in the Chinese cabbage, we compared the arrangement of introns and exons according to their phylogenetic relationships (Fig. 4B). The *BrGASA* genes in subfamily A (green) and C (pink) had 3–4 exons, whereas the *BrGASA* genes in subfamily B (blue) had 1–2 exons. The number of exons of the *BrGASA* gene in the same subfamily was similar, which indicated that the structure of these genes was conserved and that there was a close evolutionary relationship between genes [28, 40]. Subfamily A (green) was more conserved in gene structure than the other subfamilies, which indicated that the production rate of introns in these genes was higher at the early stage of evolution stage [54]. Other subfamilies might have acquired exon during evolution, resulting in the difference in the number of exon-intron. In studies of other species, the number and structure of exons in the same gene subfamily also exhibited similarities, such as in apple, grape, poplar, and potato [23, 27, 28, 55]. On the other hand, in monocotyledonous wheat, the *GASA* gene had the same number (2–4) and structure of exons [40]. These results further verified the homology in the phylogenetic analysis. The predicted motifs of BrGASA proteins were highly conserved with three structural motifs at the C-terminus, consisting of motifs 1, 2, and 3 (Fig. 4C). Homologous *BrGASA* genes also had similar protein structures, such as *BrGASA8*/*BrGASA12* in subfamily B; and *BrGASA7*/*BrGASA15*, *BrGASA6*/*BrGASA13* in subfamily C. Four and 10 motifs were found in apple and poplar GASA proteins, respectively, and proteins from the same subfamily had similar length and number [23, 28]. In rubber tree, GASA proteins had 10 motifs, of which three motifs existed in all proteins, whereas some proteins had specific motifs, indicating differences in protein function [24].

### Evolutionary relationships and collinearity analysis among *GASA* genes

**Fig. 5:**
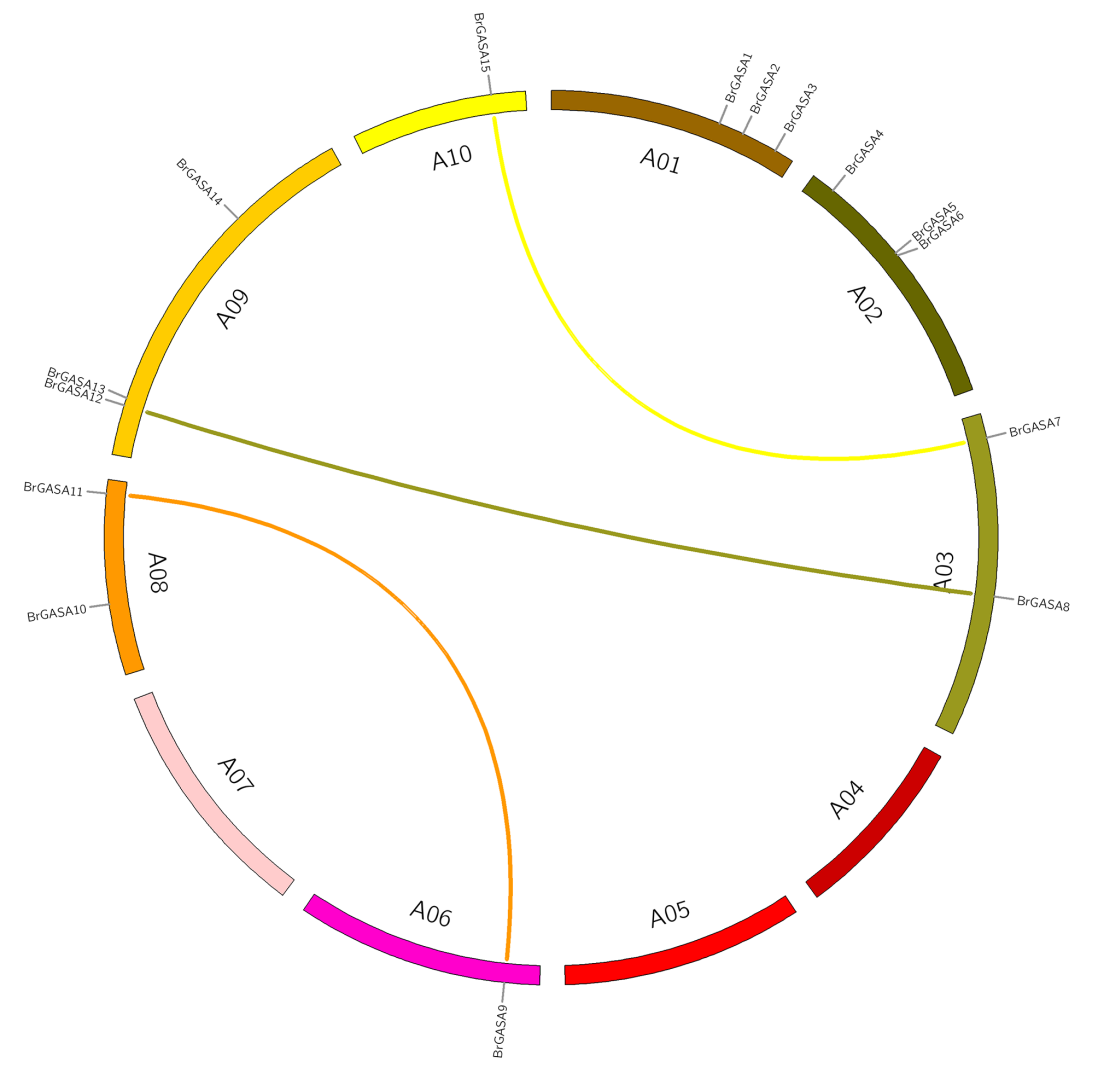
Chromosomal distribution and synteny analysis of *BrGASA* genes. Syntenic regions and chromosomal regions are depicted in different colors

**Fig. 6:**
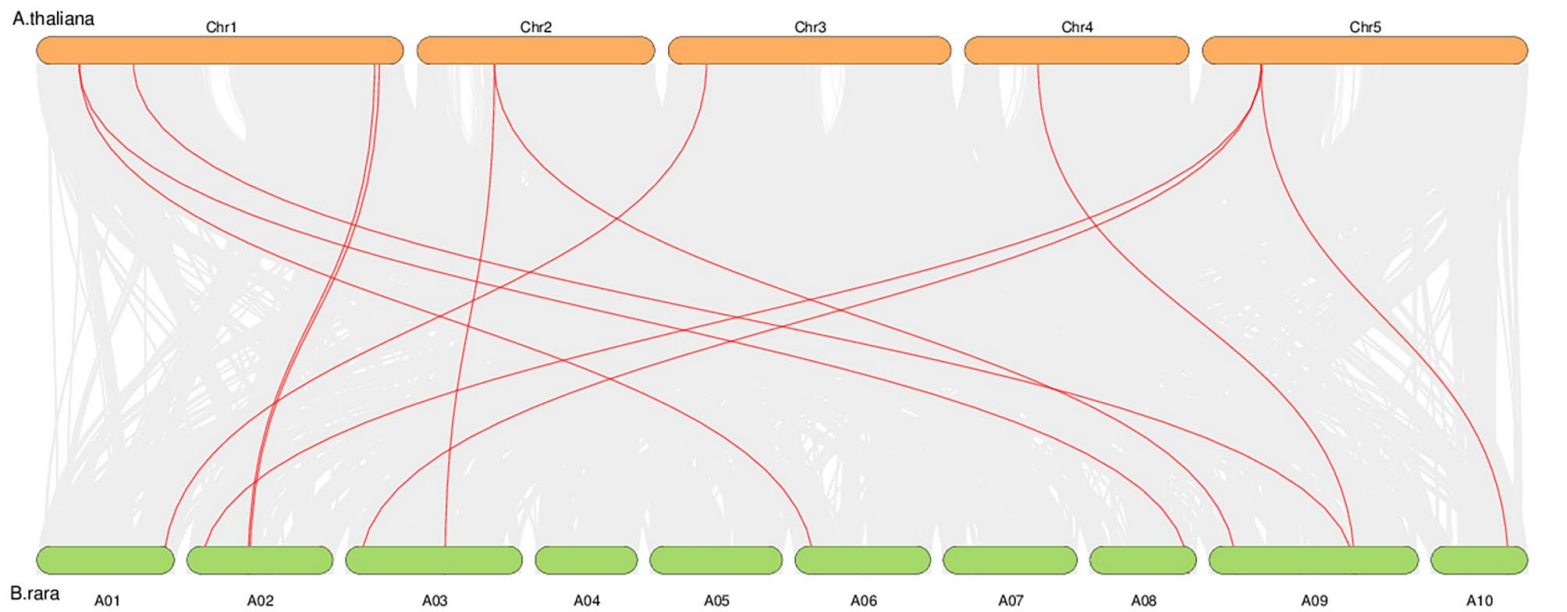
Synteny analysis of *GASA* genes between Chinese cabbage and *A. thaliana*. Grey lines in the background indicate collinear blocks in Chinese cabbage and *A. thaliana* genomes, while the red lines highlight syntenic *GASA* gene pairs

According to our results, the 15 *BrGASA* genes were randomly distributed on 7 of the 10 chromosomes of Chinese cabbage (Fig. 1). Three whole-genome duplication (WGD) pairs of *BrGASA* genes (*BrGASA7*/*15*, *BrGASA8*/*12*, and *BrGASA9*/*11*) were distributed across five chromosomes (Fig. 5, Table S1). Synteny analysis of the *GASA* genes in the Chinese cabbage and *Arabidopsis* genomes showed that most of the *GASA* genes in the Chinese cabbage were homologous to those in the *Arabidopsis genome* (Fig. 6, Table S2). Most homologous genes on the seven chromosomes hosting the *GASA* genes were located on chromosome 1 of *Arabidopsis* (five genes), followed by chromosome 5 (three genes). Finally, two genes were located on chromosome 2, with one homologous gene on chromosomes 3 and 4.

Gene replication promotes the evolution of the genome and genetic system as well as the diversity of gene structure and function in the gene family [56]. Four gene replication modes can help in the evolution of gene families, namely: WGD, tandem duplication, segmental duplication, and transposition duplication (TRD) [57]. To illustrate the expansion pattern of *BrGASA* genes, we analyzed duplication events in the genomes of Chinese cabbage (Table 3). Six genes made up three WGD pairs (*BrGASA7*/*15*, *BrGASA8*/*12*, and *BrGASA9*/*11*), which indicated that they all had a common ancestor. In addition to WGD duplication, four pairs of TRD, *BrGASA10*/*5*, *BrGASA13*/*6*, *BrGASA1*/*5*, and *BrGASA2*/*8* were observed among seven genes. This indicates that both WGD and TRD contributed to expanding of the *GASA* gene family in the Chinese cabbage. In general, WGD is believed to contribute significantly toward the evolution of morphological and physiological diversity, and TRD is important within the context of single-gene replication [58]. New genes evolve through selection and mutation [59]. Studies on other species have shown that the *GASA* gene in wheat underwent large scale duplication or tandem duplication events [40]. The subfamily of cotton *GASA* genes that has underwent WGD replication was highly conserved during evolution [29]. Segmental duplication was a common replication method in the GASA gene family, which has been found in the results of apple, soybean, and grape [23, 26, 27]. The ratio of non-synonymous (Ka) to synonymous (Ks) can be used to describe the evolution history [60]. In our study, the Ka/Ks value of each repeat pair was much smaller than 1, indicating that these genes were selected for purification [56]. Similarly, only one pair of homologous genes in wheat had a Ka/Ks value greater than 1, and most genes were in the purifying selection [40]. In conclusion, environmental changes had little impact on the *GASA* gene family evolution in Chinese cabbage. In addition, the average Ka/Ks values were 0.162 and 0.318 for the TRD- and WGD-duplicated gene pairs, respectively, indicating that TRD-duplicated genes could be more conserved.

**Table 3:** Duplications of *GASA* genes in Chinese cabbage

Gene1	Gene2	Duplication	Ka	Ks	Ka/Ks	Selection Pressure
*BrGASA10*	*BrGASA5*	TRD	0.4282	2.9957	0.1429	Purifying selection
*BrGASA13*	*BrGASA6*	TRD	0.0193	0.0757	0.2547	Purifying selection
*BrGASA1*	*BrGASA5*	TRD	0.3971	2.8870	0.1376	Purifying selection
*BrGASA2*	*BrGASA8*	TRD	0.2424	2.1314	0.1137	Purifying selection
*BrGASA7*	*BrGASA15*	WGD	0.1313	0.26849	0.4889	Purifying selection
*BrGASA8*	*BrGASA12*	WGD	0.0523	0.2345	0.2229	Purifying selection
*BrGASA9*	*BrGASA11*	WGD	0.1151	0.47622	0.2416	Purifying selection

TRD: transposition duplication; WGD: whole-genome duplication

### Cis‑acting element analysis of *BrGASA* genes in the Chinese cabbage

**Fig. 7:**
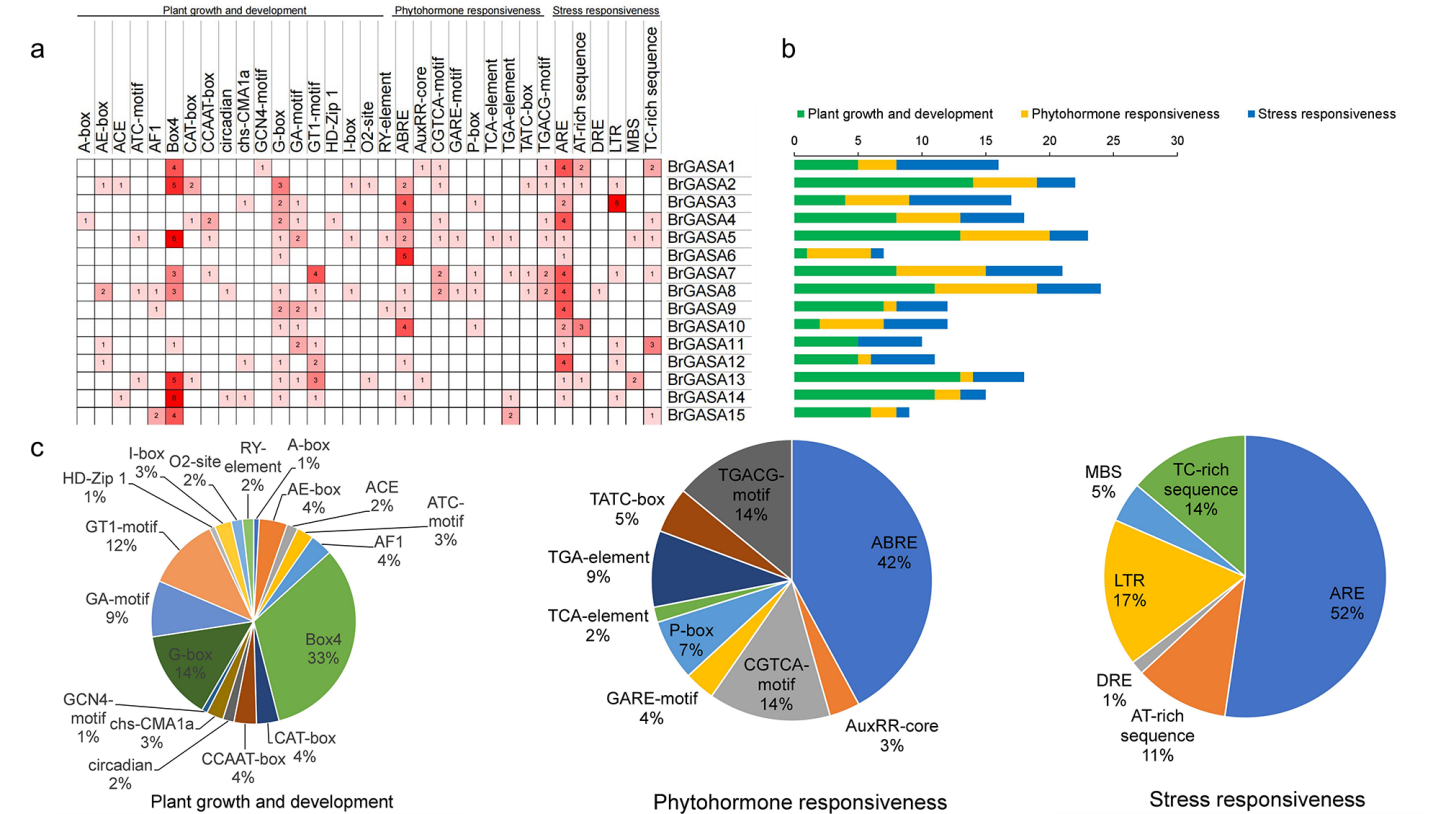
Cis-acting elements of *GASA* genes in Chinese cabbage. (**a**) Numbers and gradient red colors indicate the number of cis-acting elements in each gene; (**b**) Color-coded histograms indicate the number of identified cis-acting elements in each gene according to three categories; (**c**) Pie charts showing the proportion of different cis-acting elements in each category

Cis-acting elements in gene promoter regions can help us explore gene function [29]. To predict the potential biological function of the *GASA* genes in Chinese cabbage, we analyzed the cis-acting elements of their promoter (2 kb upstream). As shown in Fig. 7, the identified cis-acting elements were divided into three categories: plant growth and development, phytohormone responses, and stress responses. This indicated that *BrGASA* genes might participate in and affect these three biological activities. These three types of elements were also common in the GASA gene promoter sequences of other species [23, 26–29, 40, 52]. The cis-acting elements detected in most *BrGASA* gene promoters associated with plant growth and development were light-responsive elements, such as Box4 and G-box, indicating that GASA proteins might participate in light response in Chinese cabbage. Light responsive elements were the main components on the GASA gene promoter in *Glycine max* [26]. In addition to light responsive elements, there were other elements related to growth and development in some genes, including the CAT-box related to meristem expression, GCN4-motif related to endosperm expression, and HD-Zip1 related to the differentiation of palisade mesophyll cells. In a previous study, endosperm (AAGAA motif) expression and meristem activation (CCGTCC box) elements were found in grape *GASA* genes [27]. Meanwhile, we found that Chinese cabbage *GASA* genes all contain hormone-responsive elements, mainly abscisic acid (ABA) and methyl jasmonate (MeJA) elements. On the other hand, some genes contained elements related to the GA and salicylic acid (SA) responses, which may be because *BrGASA genes* are involved in the signaling pathways of these hormones [40]. In *Arabidopsis*, the most common hormone responsive elements on *GASA* genes were GA (GARE) and ABA (ABRE) [52]. Additionally, the *BrGASA* genes had rich stress response element, which was similar to the fact that the *GASA* gene family was involved in stress response research in other species. For example, in the rice *GASA* gene, elements that respond to low temperature (LTR) and drought (MBS) were the most common [40]. Among the stress response elements, all genes except *BrGASA15* had ARE elements related to anaerobic induction. Six genes contained LTR elements related to low temperature stress response and TC-rich repeat elements related to defense and stress response. AT-rich sequences were detected in four genes. *BrGASA5* and *BrGASA13* also contained drought-inducible MBS. DRE related to dehydration, low temperature, and salt stress were found only in *BrGASA8*. The above mentioned stress response elements were also present in the GASA gene promoter regions of apple, *Glycine max*, and grape [23, 26, 27].

### Expression Patterns of *GASA* genes in different tissues of Chinese cabbage

**Fig. 8:**
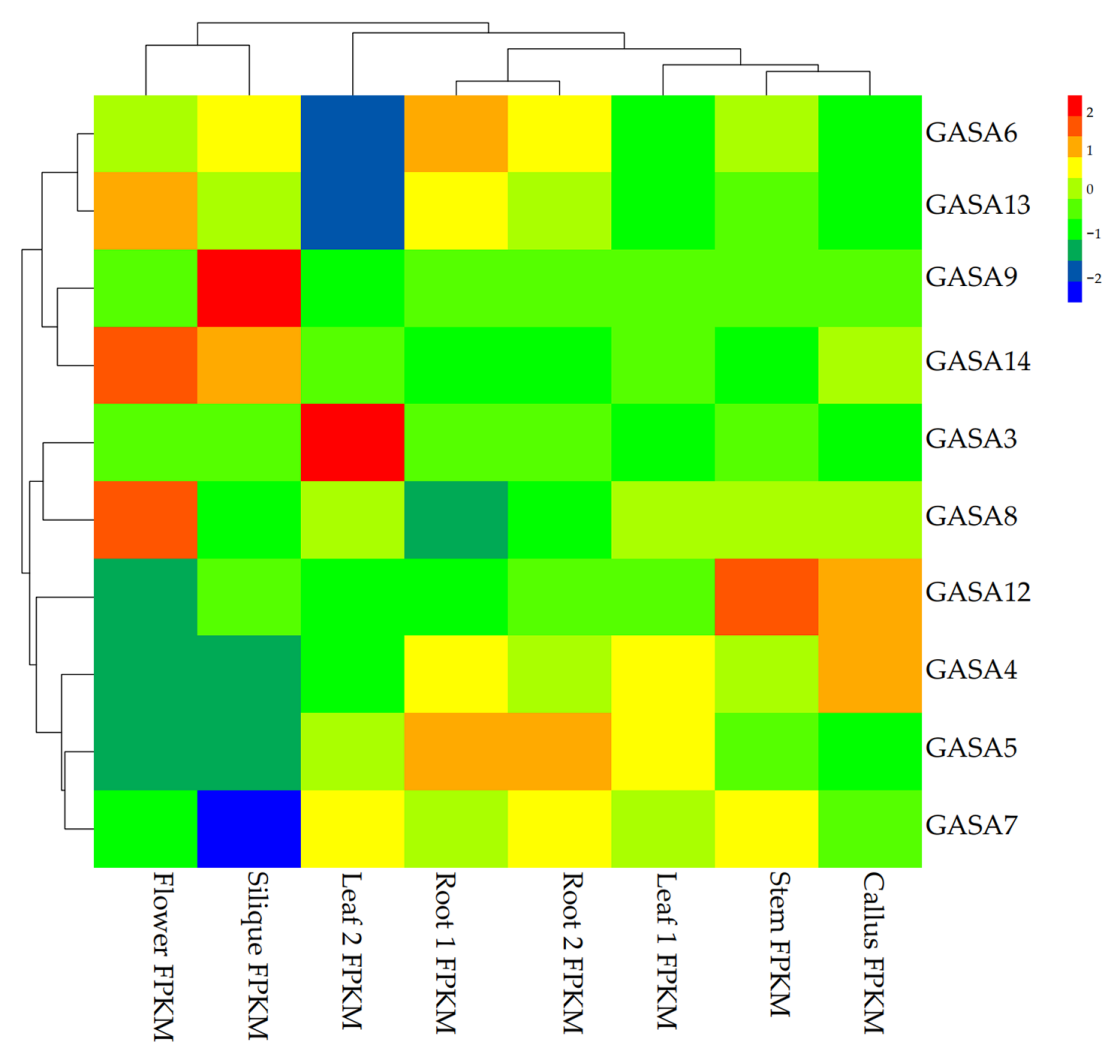
Heat map representation and hierarchical clustering of *BrGASA* genes in eight different Chinese cabbage tissues

The expression profiles of genes in different plant organs and parts can provide clues to their respective functions [33, 42]. We then investigated the expression of each *BrGASA* gene using published RNA-seq data from six different Chinese cabbage tissues during vegetative and reproductive development. The expression levels of each gene were normalized using the FPKM method. Among the 15 *GASA* genes in Chinese cabbage, the transcription levels (FPKM value) of 10 genes were determined in each tissue sample (Fig. 8). The expression of the other five *BrGASA* genes (*BrGASA1*, *BrGASA2*, *BrGASA10*, *BrGASA11*, and *BrGASA15*) was not detected in the RNA-seq libraries, which might be because they have no expression or spatiotemporal modes. In our study, *BrGASA13*, *BrGASA14*, and *BrGASA8* were highly expressed in the flowers. *BrGASA6*, *BrGASA14*, and *BrGASA9* were highly expressed in the siliques. *BrGASA5* and *BrGASA6* were highly expressed in the roots. *BrGASA4* and *BrGASA12* were highly expressed in the callus. *BrGASA12* was highly expressed in the stems. *BrGASA6*, *BrGASA13*, *BrGASA14*, *BrGASA12*, *BrGASA4*, *BrGASA5*, and *BrGASA7* were highly expressed in two or more different tissues. Noteworthily, *AtGASA15* was homologous to *BrGASA3*, whereas *AtGASA15* was expressed not only in leaves, but also in stems and flowers [52], which might suggest differences in gene function. In studies of other species, the *GASA* genes in *Arabidopsis* were expressed in root, stem, leave, flower, and developing silique [52], whereas most of the *GASA* genes in poplar were expressed in stem and root [28]. All *GASA* genes in apple were expressed more in flower, leave, and fruit than in stem and seedlings [23]. The *GASA* genes in soybean were mainly expressed in flower [26], whereas those of grape were specifically expressed in seeds [27]. Some cotton *GASA* genes had higher expression levels in fiber [29]. Finally, most of the *GASA* genes in wheat were highly expressed in embryo and anther [40].

### Relative expression of nine *BrGASA* genes in Chinese cabbage

**Fig. 9:**
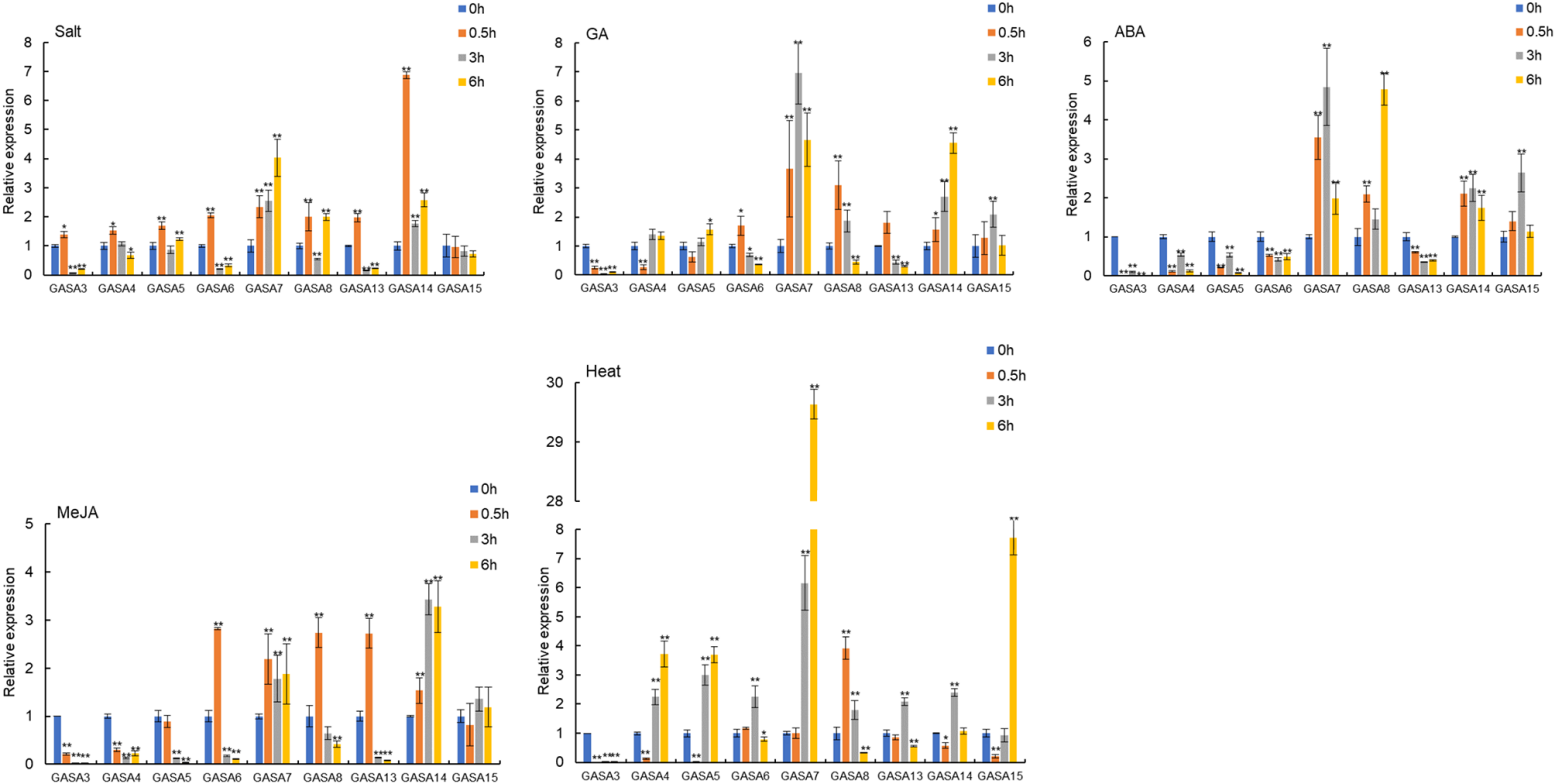
Expression profiles of nine *BrGASA* genes in response to salt, high temperature, GA3 treatment, ABA, and MeJA exposure. Quantitative reverse transcription polymerase chain reaction (qRT-PCR) analysis was used to assess the transcript levels of *BrGASA* genes in Chinese cabbage leaves sampled at 0 (baseline), 0.5, 3, and 6 h after treatment. Error bars represent the standard error of the means of three replicates. Asterisks indicate significance of the indicated differences in gene expression according to the t-test (*P < 0.05, **P < 0.01)

Next, the expression patterns of nine *BrGASA* genes were analyzed by qRT-PCR at four time points under abiotic stress and different hormonal treatments (Fig. 9). Under the salt stress of 2% NaCl, all genes except *BrGASA15* were significantly up-regulated, and the expression level of most genes was up-regulated considerably, in particular at 0.5 h time point, and then down-regulated at the last two time points. The expression level of *BrGASA14* was the highest at 0.5 h, approximately 6.8 times higher than that at baseline (0 h). The expression trends of *BrGASA7* and *BrGASA8* differed from those of the other genes. Specifically, *BrGASA7* was significantly up-regulated at 6 h after treatment, while *BrGASA8* was up-regulated at 0.5 and 6 h after treatment. Upon high temperature treatment, the expression of *BrGASA3* was significantly down-regulated, whereas the other genes were significantly up-regulated and showed differences at different time points. Only *BrGASA8* was up-regulated considerably at 0.5 h after treatment, whereas *BrGASA6*, *BrGASA13*, and *BrGASA14* were significantly up-regulated at 3 h, and *BrGASA4*, *BrGASA5*, *BrGASA7*, and *BrGASA15* were significantly up-regulated at 6 h after treatment, among which those of *BrGASA15* and *BrGASA7* were approximately 6- and 29-times greater than those at baseline, respectively.

It is important to note that the expression of *GASA* genes is controlled by GA [8]. Hormone treatment with 500 mg/L GA3 induced significant upregulation of all *BrGASA* genes, except for *BrGASA3*, which was down-regulated. Noteworthily, the expression of these genes varied in a time-dependent manner. The expression levels of *BrGASA6*, *BrGASA8*, and *BrGASA13* were significantly up-regulated at 0.5 h after treatment, whereas *BrGASA4*, *BrGASA7*, and *BrGASA15* were up-regulated 3 h after treatment, with *BrGASA7* reaching the highest expression. Only *BrGASA14* was significantly up-regulated at 6 h after treatment. In studies of other species, the *GASA* gene had also responded to exogenous GA treatment, such as *ZmGSL4*/*6* in maize [20], *TaGASR34* in wheat [40], *AtGASA14* in *Arabidopsis* [9], *HbGASA16* in rubber tree [24], and most *MdGASA* genes in apples had been down-regulated after GA treatment [23]. Several studies had shown that *GASA* was regulated by ABA [29, 34, 43]. In *Arabidopsis*, *AtGASA2/3/5/14* responded to ABA. A few *HbGASA* genes (3/16) responded to ABA [24]. In contrast, the expression of five genes (*BrGASA3*, *BrGASA4*, *BrGASA5*, *BrGASA6*, and *BrGASA13*) was significantly down-regulated following ABA treatment. *BrGASA7*, *BrGASA14*, and *BrGASA15* levels were up-regulated 3 h after treatment, of which *BrGASA7* had the highest expression. *BrGASA8* also achieved a significantly higher expression upon ABA exposure for 6 h (approximately 4.7 times higher than at baseline). The different responses induced by gibberellin and ABA may be due to these hormone antagonist effects [43]. MeJA was a natural hormone that affects the stress response of plants [61]. After treatment with MeJA, *BrGASA3*, *BrGASA4*, *BrGASA5* and *BrGASA13* were significantly down-regulated: The expression levels of *BrGASA6*, *BrGASA7*, and *BrGASA8* increased dramatically at 0.5 h after treatment and then showed a downward trend over time. However, an opposite trend was observed for *BrGASA14*, whose expression significantly increased at 3 and 6 h after treatment compared with baseline. In addition, the expression level of *BrGASA15* increased, but the difference was not statistically significant. In addition to the three hormones above, *GASA* also responded to other hormones. For example, the *MdGASA* gene in apple mainly responded to 6-BA treatment and upregulates expression [23], wheraes *HbGASA* gene in rubber tree responded to ethylene and jasmonic acid treatment [24]. These findings show that *BrGASA* is involved in and regulated by abiotic stress and hormone responses in the Chinese cabbage.

## Conclusion

In this study, 15 *GASA* genes were identified in the Chinese cabbage genome and were divided into three subfamilies. The *GASA* genes were distributed unevenly on 10 Chinese cabbage chromosomes. A total of four pairs of *GASA* genes were found to originate from tandem duplication (TRD), and three pairs of *GASA* genes originated from whole-genome duplication (WGD). Cis-acting elements of the *GASA* promoters were involved in plant development, hormonal and stress responses. The members of *GASA* genes showed differential expression patterns in diverse tissues, and differential responses were also found under different abiotic and hormonal stress. The results of our study provide valuable clues for studying of *GASA* genes in Chinese cabbage. We will further analyze the molecular mechanism of the GASA genes response to hormones and stress in Chinese cabbage, and lay the foundation for improving the cultivation, yield, and quality of Chinese cabbage through biological breeding.

## Methods

### Identification of *GASA* family genes in Chinese cabbage

*Brassica rapa* (v3.5) data were downloaded from the Chinese cabbage database (http://brassicadb.cn), and the Hidden Markov Model (HMM) profile of the *GASA* domain (PF02704) was obtained from the Pfam database (http://pfam.xfam.org/). The multi-transcript gene was filtered according to the gff gene annotation file, and the longest mRNA sequence was selected as the representative of the gene. The hmmsearch program in the HMMER (v 3.1b2) software package was used to detect protein sequences containing the *GASA* domain (PF02704). The *Arabidopsis* genome information was downloaded from the *Arabidopsis* database (http://arabidopsis.org) using Diamond (v0.9.24.125) to build a database of 15 known *GASA* family genes in *Arabidopsis* and conduct a blastp comparison to identify homologous genes. Parameter settings: e-value 1e-20. Fifteen *GASA* family genes were obtained using the abovementioned methods.

### Physicochemical Properties, phylogeny, and Synteny Analysis

All identified *BrGASA* protein, coding, and genomic sequences, as well as related information regarding the start-end position of the gene, number of amino acids, and chromosome location, were downloaded from the Chinese cabbage database. Information on the physicochemical properties of *GASA* proteins was obtained from the online ExPASy program (https://web.expasy.org/protparam/) using protein sequences [62]. In *silico* analysis of subcellular location and tertiary structure of proteins was performed using online programs: The WOLF PSORTII program (https://wolfpsort.hgc.jp/) [63] and PHYRE server v2.0 (http://www.sbg.bio.ic.ac.uk/phyre2/html/page.cgi?id=index), respectively. A phylogenetic tree was constructed using Fast Tree software based on the neighbor-joining method with a bootstrap test of 1,000 replicates. The occurrence of replication events and synteny of *GASA* genes in Chinese cabbage were analyzed and visualized using MCScanX [64], DupGen_finder [58], and TBtools [65].

### Exon–Intron, Gene structure, conserved Motif, and promoter analysis

Structural information, such as the number of introns and exons of the Chinese cabbage *GASA* gene family members, was obtained from the protein annotation files retrieved from the Chinese cabbage database. The gene structure was determined according to the corresponding sequence, and the gene structure map was generated using TBtools software (v 0.665). The MEME platform (https://meme-suite.org/meme/doc/meme.html) was used to identify conserved motifs in the BrGASA proteins [66] (default parameters with the maximum number of motifs set to 10). Furthermore, the 2 kb region upstream of the start codon of candidate *BrGASA* genes was examined for the presence of cis-elements. The PlantCARE program (http://bioinformatics.psb.ugent.be/webtools/plantcare/html/) was used to search for regulatory elements.

### Tissue-specific gene expression analysis of *BrGASA* genes

For the expression profiling of the *GASA* genes in Chinese cabbage, we utilized the Illumina RNA-Seq data previously generated and analyzed by Tong et al. [67]. Six tissues including callus, root, stem, leaf, flower, and silique of the Chinese cabbage cultivar Chiifu were studied. The transcript abundance is expressed as fragments per kilobase of exon model per million mapped reads (FPKM). The expression profiles of the Chinese cabbage *GASA* genes from each sample were clustered and a heatmap was drawn using the HemI program (http://hemi.biocuckoo.cn/). After normalization using the default linear method, the expression data were clustered using the hierarchical average linkage algorithm and the Euclidean distance similarity metric algorithm on both the horizontal and vertical axes.

### Plant Growth conditions, treatments, and Sampling

The wild-type ‘FT’ was a double haploid line obtained by microspore culture from the Chinese cabbage variety ‘Fukuda 50’, screened by Shenyang Greenstar Chinese cabbage research institute (Shenyang, China). In September 2022, the seeds stored in our laboratory were placed in a moist petri dish for germination at room temperature. Under routine management, the sprouting seeds were sown into a 32-well seedling tray in a solar greenhouse at Shenyang Agricultural University (Shenyang, China). The solar greenhouse was monitored at 41 degrees north latitude and 123.33 degrees east longitude. After sowing for four weeks, the uniform seedlings were selected and treated with a high temperature of 35 ℃, 2% NaCl, 500 mg/L GA3, 100 µmol/L ABA, 100 µmol/L MeJA and under natural drought conditions for 0 h, 0.5 h, 3 h, and 6 h [19, 28, 68]. The leaves treated with different treatments for 0 h, 0.5 h, 3 h, and 6 h were sampled, which were frozen with liquid nitrogen and stored at − 80 ℃ for the following experiment. Three biological replicates were performed for each treatment.

### qRT- PCR expression analysis

Total RNA was extracted from Chinese cabbage leaves using the RNAsimple Total RNA Kit (TIANGEN BIOTECH, Beijing, China). First-strand cDNA fragments were synthesized from total RNA using the FastKing RT Kit (TIANGEN BIOTECH, Beijing, China). Before the subsequent PCR reaction, the cDNA samples were stored at -20 ℃. The *BrGASA* CDS sequence and Primer Premier software (version 5.0) were used for primer design and synthesis by the biology company (Sangon Biotech (Shanghai, China). *BrActin* (*BraA10g027990.3 C*), was used as an internal reference gene [69]. The primer sequences are shown in Table S3. The amplification reaction contained 10 × diluted cDNA 1 µL, upstream primers 0.2 µL, downstream primers 0.2 µL, SybrGreen qPCR Master Mix 5 µL, and ddH_2_O 3.6 µL. The PCR cycling conditions included an initial polymerase activation step of 95 ℃ for 3 min, followed by 45 cycles of 95 ℃ for 15 s and 60 ℃ for 30 s. Three biological replicates were performed for each sample. The relative expression levels of the *BrGASA* gene are represented in the form of relative changes by the 2^−∆∆Ct^ method [70]. The statistical analysis was performed using Microsoft Excel 2019 and SPSS 26.0.

### Electronic Supplementary Material

Below is the link to the electronic supplementary material.

Supplementary Material 1

## Data Availability

The datasets supporting the conclusions of this article are included within the article and its additional files. Genomic sequences and gene annotation information of *Brassica rapa* were downloaded from http://brassicadb.cn/#/.
