# Supplementary material for: Genome-wide identification and expression analysis of the GASA gene family in Chinese cabbage (Brassica rapa L. ssp. pekinensis)
Source: BMC Genomics. 2023 Nov 6;24:668. doi: 10.1186/s12864-023-09773-9 (PMC10629197; doi:10.1186/s12864-023-09773-9)
Supplement: Supplementary file 1 — Supplementary Material 1 [file 12864_2023_9773_MOESM1_ESM.docx]

**Table S1** Synteny blocks of the *GASA* genes within the Chinese cabbage genome.

| **Gene in the syteny region** | | **Region 1** | | | **Region 2** | | |
| --- | --- | --- | --- | --- | --- | --- | --- |
| **Gene 1** | **Gene 2** | **Chr** | **Start** | **Stop** | **Chr** | **Start** | **Stop** |
| *BrGASA7* | *BrGASA15* | A03 | 2834280 | 2835698 | A10 | 16685009 | 16686428 |
| *BrGASA8* | *BrGASA12* | A03 | 21432680 | 21433470 | A09 | 5878441 | 5879203 |
| *BrGASA9* | *BrGASA11* | A06 | 4145012 | 4145212 | A08 | 21375636 | 21376007 |

**Table S2** *GASA* homologous genes in Chinese cabbage and *Arabidopsis.*

| **Gene name** | **homologous gene** | **E_Value** |
| --- | --- | --- |
| *BraA01g041680.3.5C* | *AT3G10185* | 2e-12 |
| *BraA02g005980.3.5C* | *AT5G15230* | 1e-50 |
| *BraA02g023240.3.5C* | *AT1G74670* | 2e-53 |
| *BraA02g023850.3.5C* | *AT1G75750* | 3e-46 |
| *BraA03g006590.3.5C* | *AT5G14920* | 3e-12 |
| *BraA03g043210.3.5C* | *AT2G14900* | 4e-34 |
| *BraA06g007530.3.5C* | *AT1G10588* | 2e-25 |
| *BraA08g032610.3.5C* | *AT1G10588* | 3e-32 |
| *BraA09g010560.3.5C* | *AT2G14900* | 9e-37 |
| *BraA09g042130.3.5C* | *AT1G22690* | 3e-24 |
| *BraA09g042130.3.5C* | *AT4G09610* | 4e-14 |
| *BraA10g024440.3.5C* | *AT5G14920* | 1e-12 |

**Table S3** Primer sequences used in expression analysis of the *GASA* genes in Chinese cabbage.

| **Gene** | **Forward and reverse primer sequence (5'~3')** | **Fragment length** | **melting temperature** | **GC content** |
| --- | --- | --- | --- | --- |
| *BrGASA3* | F AACCACATCCACCACAGTCC  R AGTTGTTGTAGCAAGGGCAGA | 234bp | 57.2℃  57.6℃ | 55.0%  47.6% |
| *BrGASA4* | F CGTTGTCTTCTCCTTGCTTCA  R TGTCTGGCTACACCTCCTATCAC | 139bp | 58.0℃  58.6℃ | 47.6%  52.2% |
| *BrGASA5* | F TCATACCAATGTGGAGGGGA  R TTGTTTGTTGCCGTAAGTGC | 132bp | 58.1℃  57.2℃ | 50.0%  45.0% |
| *BrGASA6* | F ATCTCTTCTTGTTCTCCAACTTGTC  R ATACACGCACTTCCACAATCAAT | 96bp | 58.2℃  59.0℃ | 40.0%  39.1% |
| *BrGASA7* | F CCACCCGTCAACCCAATC  R GCCAGGAGGAACGCACTT | 204bp | 58.6℃  58.0℃ | 61.1%  61.1% |
| *BrGASA8* | F CATCCCTTGCTTCGGCTAC  R GCTTGTTCCCATAAGTTCCTGA | 210bp | 58.1℃  58.7℃ | 57.9%  45.5% |
| *BrGASA13* | F TATCTTTTCTTGTTCTCCAACTCGT  R AGCCTGCACCGTGCTATACA | 121bp | 59.0℃  59.1℃ | 36.0%  55.0% |
| *BrGASA14* | F GAGGAGAACCAAGTGGAGGC  R CGGAGATTAGCGTAGCAAGG | 206bp | 58.4℃  58.0℃ | 60.0%  55.0% |
| *BrGASA15* | F ACCTGTGAACCCAATCCCAT  R AGGCACGCAATCTAACCGA | 115bp | 58.7℃  59.0℃ | 50.0%  52.6% |
| *BrActin* | F ATCTACGAGGGTTATGCT  R CCACTGAGGACGATGTTT | 410bp | 46.3℃  50.0℃ | 44.4%  50.0% |

All primers have no hairpin structure. Cross dimer was only found in *BrGASA6* and *BrGASA13*.
